# Particularités du syndrome inflammatoire multisystémique de l´adulte lié au SARS-CoV-2

**DOI:** 10.11604/pamj.2025.52.29.47987

**Published:** 2025-09-18

**Authors:** Fatma Medhioub Kaaniche, Farah Zouari, Salma Jerbi, Ines Dahech, Arij Abdellatif, Yoser Ben Taher, Wiem Feki, Zina Hakim, Sondes Briki, Dorsaf Dlensi, Rania Allala

**Affiliations:** 1 Service Universitaire de Réanimation, Hôpital Régional Mahres, Faculté de Médecine de Sfax, Université de Sfax, Sfax, Tunisie,; 2 Service de Radiologie, Centre Hospitalier Universitaire Hédi Chaker, Faculté de Médecine de Sfax, Université de Sfax, Sfax, Tunisie,; 3 Unité de Médecine de Travail, Hôpital Régional Mahres, Faculté de Médecine de Sfax, Université de Sfax, Sfax, Tunisie,; 4 Service de Maxillofacial, Centre Hospitalier Universitaire Habib Bourguiba, Faculté de Médecine de Sfax, Université de Sfax, Sfax, Tunisie

**Keywords:** Syndrome inflammatoire multisystémique, adulte, COVID-19, MIS-A, immunoglobulines, Multisystem inflammatory syndrome, adult, COVID-19, MIS-A, immunoglobulins

## Abstract

Le syndrome inflammatoire multisystémique de l'adulte (MIS-A) est une entité rare et sévère survenant après une infection à Severe Acute Respiratory Syndrome Coronavirus 2 (SARS-CoV-2), souvent méconnue chez l'adulte. Nous décrivons les caractéristiques cliniques, paracliniques, thérapeutiques et pronostiques du MIS-A à travers une revue structurée de la littérature. Une recherche a été menée dans les bases de données PubMed, Scopus et Web of Science jusqu'en mai 2024. Les articles inclus décrivaient des cas cliniques ou des séries de cas de MIS-A chez l'adulte. Dix-huit (18) articles ont été inclus. Le MIS-A se manifeste principalement par une fièvre persistante, une atteinte multiviscérale, une inflammation biologique marquée et une Polymerase Chain Reaction (PCR) SARS-CoV-2 souvent négative mais une sérologie positive. Le traitement repose sur les immunoglobulines, les corticostéroïdes et parfois les anti-IL-6. Bien que rare, le MIS-A représente une urgence médicale à considérer dans les suites d'une infection à coronavirus disease (COVID-19), même asymptomatique. Son diagnostic repose sur des critères cliniques et biologiques non spécifiques, rendant sa reconnaissance difficile. Un traitement immunomodulateur précoce permet d'améliorer le pronostic.

## Abstract (en)

Multisystem inflammatory syndrome in adults (MIS-A) is a rare and severe entity occurring after SARS-CoV-2 infection, and it is often underrecognized in adults. The purpose of this study is to describe the clinical, paraclinical, therapeutic, and prognostic characteristics of MIS-A through a structured review of the literature. A search was conducted in PubMed, Scopus, and Web of Science databases up to May 2024. Articles included were clinical case reports or case series of MIS-A in adults. Eighteen (18) articles were included. MIS-A mainly manifests as persistent fever, multiorgan involvement, marked inflammatory response, and frequently negative SARS-CoV-2 PCR but positive serology. Treatment is based on immunoglobulins, corticosteroids, and, in some cases, anti-IL-6 therapy. Although rare, MIS-A represents a medical emergency to be considered in the aftermath of COVID-19 infection, even in asymptomatic cases. Diagnosis is based on nonspecific clinical and biological criteria, which makes recognition challenging. Early immunomodulatory treatment can improve prognosis.

## Introduction

Le MIS-A est une complication post-infectieuse rare du SARS-CoV-2. Décrit pour la première fois au début de la pandémie de COVID-19, il partage certaines caractéristiques avec le *Multisystem inflammatory syndrome in children* (MIS-C) chez l'enfant, mais son profil clinique reste moins bien compris. Le syndrome inflammatoire multisystémique de l'adulte (MIS-A) survient généralement dans les semaines suivant une infection à coronavirus, et se manifeste par une inflammation systémique multiviscérale, souvent sévère. L'objectif de cette revue est de fournir une synthèse des données publiées sur le MIS-A associé au SARS-CoV-2. Elle vise à décrire les caractéristiques cliniques, biologiques et radiologiques rapportées dans la littérature, ainsi que les critères diagnostiques actuellement proposés. Elle analyse également les différentes stratégies thérapeutiques utilisées et leurs résultats, tout en discutant du pronostic et de l'évolution des patients.

## Méthodes

**Sources de données et stratégie de recherche:** une recherche systématique de la littérature a été réalisée dans les bases de données PubMed, Scopus et Web of Science, couvrant la période de janvier 2020 à avril 2024. Les recherches ont été effectuées à l'aide d'opérateurs booléens (AND/OR) et ont été limitées aux études humaines en anglais et en français.

### Critères d'inclusion et d'exclusion

**Inclusion:** études rapportant des cas cliniquement confirmés de MIS-A, incluant des données cliniques, paracliniques ou thérapeutiques.

**Exclusion:** études sur le MIS-C exclusivement, lettres sans données cliniques exploitables, éditoriaux, études précliniques ou expérimentales.

**Figure 1:**
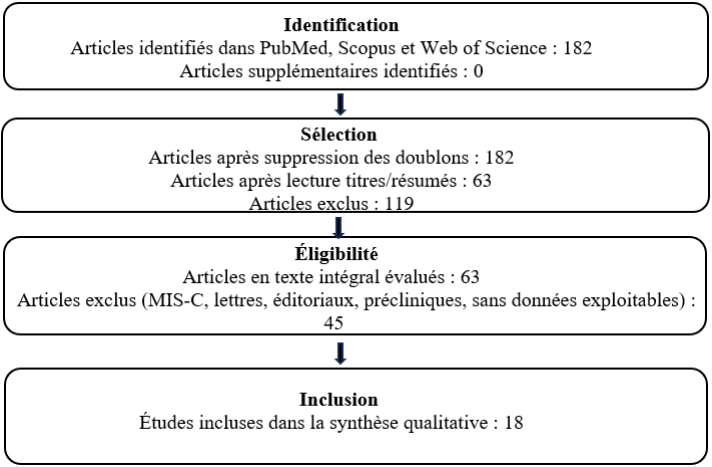
diagramme PRISMA du processus de sélection des études

**Processus de sélection des études:** deux relecteurs indépendants ont analysé les titres, les résumés puis les textes complets des articles identifiés. Les désaccords ont été résolus par consensus. Sur 182 articles identifiés initialement, 63 ont été retenus pour évaluation approfondie, et 18 ont finalement été inclus dans cette revue (Figure 1).

**Données collectées et extraction:** pour chaque article inclus, les données suivantes ont été systématiquement extraites à l'aide d'une grille standardisée: *1) caractéristiques démographiques:* âge, sexe, comorbidités. *2) caractéristiques cliniques:* signes cliniques initiaux, atteinte multiviscérale, paramètres vitaux à l'admission. *3) explorations paracliniques:* anomalies biologiques (inflammatoires, hématologiques, cardiaques, rénales, hépatiques), imagerie (radiographie thoracique, échocardiographie, scanner). *4) prise en charge thérapeutique:* recours aux corticoïdes, immunoglobulines intraveineuses, biothérapies, antiviraux, antibiotiques, anticoagulation, ventilation mécanique ou autres mesures de support. *5) évolution et pronostic:* durée d´hospitalisation, admission en soins intensifs, complications (choc, insuffisance cardiaque, atteinte rénale, respiratoire ou neurologique), issue clinique (guérison ou décès).

L'extraction des données a été effectuée indépendamment par deux investigateurs et vérifiée par un troisième afin de réduire les biais et garantir l'exhaustivité.

## Résultats

**Données épidémiologiques:** les publications disponibles concernant le MIS-A sont principalement constituées d'études de cas ou de séries cliniques. Bastug *et al*. [1] ont identifié, entre janvier 2020 et février 2021, un total de 51 patients âgés de 18 ans ou plus, atteints de MIS-A. L'âge moyen était de 30 ans, sans prédominance marquée de sexe ou d'origine ethnique. Certains présentaient des comorbidités cardiovasculaires ou respiratoires, tandis que d'autres étaient auparavant en bonne santé.

**Manifestations cliniques:** la physiopathologie du MIS-A reste sujette à hypothèse, la plus admise étant celle d'une dérégulation immunitaire post-infectieuse. Le tableau cliniquement observé associe des caractéristiques du syndrome de Kawasaki, du syndrome d'activation macrophagique et du syndrome de choc toxique [2-6]. Le début des symptômes survient généralement entre deux et cinq semaines après une infection à COVID-19, parfois asymptomatique. Néanmoins, des présentations concomitantes à une infection aiguë ont également été rapportées [7]. La fièvre est presque constante [8-12]. Elle s'accompagne fréquemment d'asthénie, de dyspnée, de douleurs thoraciques, d'hypotension, de tachycardie, ainsi que de signes gastro-intestinaux (nausées, vomissements, diarrhée). Un exanthème diffus, une conjonctivite bilatérale et une polyadénopathie sont aussi rapportés [13-15] (Tableau 1).

**Tableau 1:** caractéristiques démographiques, antécédents médicaux et manifestations cliniques des adultes atteints de MIS-A inclus dans la revue systématique (n = 8 patients, publiés entre 2020 et 2024)

	Sexe/Age	Antécédents	Présentation clinique
Moghadam *et al*. [8]	Masculin/21 ans	-	Fièvre, oppression thoracique, diarrhée sanglante, macules rondes érythémateuses, conjonctivite
Ahsan *et al*. [12]	Masculin/28 ans	Béta-thalassémie	Fièvre, fatigue, myalgie, nausées, vomissements, éruption généralisée, conjonctivite
Fox *et al*. [14]	Féminin/31 ans	Obésité, diabète, hypertension artérielle	Fièvre, dyspnée, cervicalgies, manifestations digestives
Kofman *et al*. [15]	Féminin/25 ans	-	Fièvre, dyspnée, toux, adénopathies, manifestations digestives, état de choc
Chau *et al*. [16]	Masculin/20 ans	-	Fièvre, dyspnée, myalgie, cervicalgies, manifestations digestives
Chau *et al*. [16]	Masculin/33 ans	Alcoolisme chronique	Fièvre, dyspnée, symptômes gastro-intestinaux, éruption cutanée
Chau *et al*. [16]	Masculin/34 ans	-	Fièvre, dyspnée, douleurs thoraciques, manifestations digestives, éruption cutanée
Chau *et al*. [16]	Masculin/42 ans	-	Fièvre, douleurs thoraciques, toux, éruption cutanée

**Données paracliniques:** le MIS-A se caractérise par une inflammation systémique intense: élévation des protéines de phase aiguë (C-reactive protein (CRP), procalcitonine, ferritine), hypercoagulabilité (augmentation des D-dimères), et atteinte myocardique (élévation de la troponine et du BNP). Une atteinte rénale ou hépatique peut également être observée [1]. Face à la fréquence de tests PCR négatifs chez ces patients, la société américaine des maladies infectieuses recommande de coupler la recherche du virus à celle des anticorps [16]. La positivité des tests sérologiques, associée à un syndrome inflammatoire marqué, constitue un argument fort en faveur du MIS-A [17].

Sur le plan radiologique, des images thoraciques d'opacités en verre dépoli sont fréquemment notées. L'électrocardiogramme peut révéler une tachycardie sinusale, des troubles du rythme ou des anomalies du segment ST ou de l'onde T. L'échocardiographie met parfois en évidence une dysfonction ventriculaire gauche ou droite [7].

**Prise en charge thérapeutique:** le traitement repose sur l'administration d'immunoglobulines à forte dose, de corticostéroïdes et d'inhibiteurs de l'interleukine 6, visant à moduler la réponse inflammatoire et à prévenir les complications cardiaques, notamment les anévrismes coronaires. Les formes graves nécessitent une hospitalisation en soins intensifs, avec recours aux amines vasoactives en cas d'état de choc. L'intubation, la ventilation mécanique ou l'oxygénation par membrane extracorporelle (ECMO) peuvent être indiquées dans les cas d'insuffisance hémodynamique ou respiratoire [7].

## Discussion

Le MIS-A représente une complication post-infectieuse rare mais potentiellement grave du SARS-CoV-2. Bien que le MIS-C soit aujourd'hui bien caractérisé chez l'enfant, le MIS-A reste moins connu, avec une compréhension encore incomplète de sa physiopathologie, de ses critères diagnostiques et de son pronostic à long terme. Sur le plan pathogénique, le MIS-A semble résulter d'une réponse immunitaire exacerbée, survenant de manière différée après une infection aiguë au SARS-CoV-2, même lorsqu'elle est cliniquement silencieuse. Cette hypothèse est soutenue par le délai de survenue des symptômes, généralement entre 2 et 5 semaines après l'infection initiale [7], et la fréquence élevée de sérologies SARS-CoV-2 positives en l'absence de PCR détectable [16,17]. Cela suggère un phénomène d'activation immunitaire secondaire, probablement médié par les cytokines et les auto-anticorps.

Cliniquement, le MIS-A regroupe des manifestations variées, simulant plusieurs syndromes inflammatoires systémiques: Kawasaki-like, syndrome d'activation macrophagique ou encore choc toxique [2-6]. Cette hétérogénéité contribue au retard diagnostique, d'autant plus que les symptômes initiaux, fièvre, dyspnée, signes digestifs, éruption cutanée, conjonctivite, peuvent être attribués à d'autres causes infectieuses ou auto-immunes [8-15]. Les atteintes myocardiques, fréquemment décrites (élévation de la troponine, anomalies ECG, dysfonction ventriculaire) [7], soulignent le risque de décompensation hémodynamique, parfois fatale si le traitement n'est pas instauré rapidement.

L'un des défis majeurs reste l'absence de critères diagnostiques unifiés. Bien que les Centers for Disease Control (CDC) et l'Organisation mondiale de la Santé (OMS) aient proposé des définitions provisoires, celles-ci varient selon les pays et les institutions. La négativité de la PCR dans de nombreux cas impose une prise en compte systématique de la sérologie dans le bilan étiologique des syndromes inflammatoires inexpliqués post-COVID-19 [16]. Une autre limite est la rareté des études prospectives : la majorité des données provient de séries de cas ou d'observations isolées, ce qui limite la généralisation des résultats [1].

Sur le plan thérapeutique, le consensus repose actuellement sur l'utilisation d'immunoglobulines polyvalentes intraveineuses à forte dose, de corticostéroïdes et d'immunomodulateurs comme les antagonistes de l'interleukine 6 (ex. tocilizumab). Cette stratégie s'inspire du traitement du MIS-C et de la réponse au COVID-19 sévère. L'efficacité de cette approche a été rapportée dans plusieurs cas, avec une amélioration rapide de l'état hémodynamique et de la biologie inflammatoire [7,10,15]. Toutefois, l'absence d'études contrôlées ne permet pas encore de déterminer la meilleure séquence thérapeutique ni les indications précises des biothérapies.

Enfin, le suivi à long terme des patients ayant présenté un MIS-A reste mal documenté. Certaines publications rapportent des séquelles cardiaques persistantes, en particulier des dysfonctions myocardiques et des anévrismes coronaires [13,15], soulignant la nécessité d'un suivi spécialisé prolongé.

**Limites:** cette revue narrative présente plusieurs limites: i) l'absence de protocole d'enregistrement préalable, ii) le caractère descriptif des études incluses, iii) l'hétérogénéité des critères diagnostiques d'un article à l'autre.

## Conclusion

Le syndrome inflammatoire multisystémique de l'adulte post-COVID-19 est une entité grave mais potentiellement réversible, à condition d'un diagnostic rapide et d'un traitement adapté. Son évocation doit être systématique devant une inflammation inexpliquée chez l'adulte ayant eu une infection récente au SARS-CoV-2. Une prise en charge précoce par immunoglobulines et corticostéroïdes peut prévenir les complications graves, en particulier cardiaques.

### Etat des connaissances sur le sujet

- Le syndrome inflammatoire multisystémique est bien décrit chez l'enfant (MIS-C), mais reste rare et encore mal compris chez l'adulte (MIS-A);
- Le MIS-A survient après une infection récente par le SARS-CoV-2, souvent avec une PCR négative mais une sérologie positive;
- Les manifestations cliniques sont variables mais souvent sévères, avec une atteinte multiviscérale et des complications cardiovasculaires.

### Contribution de notre étude à la connaissance

- Le MIS-A touche principalement les jeunes adultes (20-42 ans), souvent en bonne santé, avec quelques comorbidités signalées comme l'obésité, le diabète ou l'alcoolisme;
- La fièvre est constante, accompagnée fréquemment de symptômes digestifs, respiratoires et de douleurs thoraciques, tandis que des manifestations cutanéo-muqueuses apparaissent dans 40 à 60 % des cas;
- Les examens biologiques révèlent une inflammation intense et des atteintes cardiaques fréquentes, avec une PCR SARS-CoV-2 souvent négative mais une sérologie positive dans environ 80 % des cas, et la prise en charge repose sur les immunoglobulines intraveineuses et les corticostéroïdes, conduisant à une amélioration rapide de l'état clinique et biologique.
